# Reconstructing grassland fire history using sedimentary charcoal: Considering count, size and shape

**DOI:** 10.1371/journal.pone.0176445

**Published:** 2017-04-27

**Authors:** Berangere A. Leys, Julie L. Commerford, Kendra K. McLauchlan

**Affiliations:** Department of Geography, Kansas State University, Manhattan, Kansas, United States of America; Ecole Pratique des Hautes Etudes, FRANCE

## Abstract

Fire is a key Earth system process, with 80% of annual fire activity taking place in grassland areas. However, past fire regimes in grassland systems have been difficult to quantify due to challenges in interpreting the charcoal signal in depositional environments. To improve reconstructions of grassland fire regimes, it is essential to assess two key traits: (1) charcoal count, and (2) charcoal shape. In this study, we quantified the number of charcoal pieces in 51 sediment samples of ponds in the Great Plains and tested its relevance as a proxy for the fire regime by examining 13 potential factors influencing charcoal count, including various fire regime components (*e*.*g*. the fire frequency, the area burned, and the fire season), vegetation cover and pollen assemblages, and climate variables. We also quantified the width to length (W:L) ratio of charcoal particles, to assess its utility as a proxy of fuel types in grassland environments by direct comparison with vegetation cover and pollen assemblages. Our first conclusion is that charcoal particles produced by grassland fires are smaller than those produced by forest fires. Thus, a mesh size of 120μm as used in forested environments is too large for grassland ecosystems. We recommend counting all charcoal particles over 60μm in grasslands and mixed grass-forest environments to increase the number of samples with useful data. Second, a W:L ratio of 0.5 or smaller appears to be an indicator for fuel types, when vegetation surrounding the site is before composed of at least 40% grassland vegetation. Third, the area burned within 1060m of the depositional environments explained both the count and the area of charcoal particles. Therefore, changes in charcoal count or charcoal area through time indicate a change in area burned. The fire regimes of grassland systems, including both human and climatic influences on fire behavior, can be characterized by long-term charcoal records.

## Introduction

Fire is one of the key Earth system processes acting on vegetation composition, carbon stocks, and nutrient cycling [1]. Frequent fire is particularly important for maintaining grassland ecosystems, defined recently as “non-wetland type with at least 10% vegetation cover, dominated or co-dominated by graminoid and forb growth forms, and where the trees form a single-layer canopy with either less than 10% cover and 5 m height (temperate) or less than 40% cover and 8 m height (tropical)” [2]. High frequency fires, such as those occurring annually, maintain grassland ecosystems through detrimental effects on tree biomass [3–5]. Currently, grassland systems encompass 80% of Earth’s fire activity each year despite representing 40% of terrestrial land cover [6]. The majority of grassland fires occur in Africa and Australia, but extensive grassland fires occur in the Americas and Asia due to both human activities [7] and wildfires [8]. In North America, many grasslands are managed by prescribed fires, but the frequency of burning needed to restore or maintain ecosystem services and biodiversity remains based on experiments [9,10]. The fire history of this region is largely unknown due to challenges with each of the three approaches used for reconstructions: sedimentary charcoal [11], dendrochronological [12], and historical records [13].

Fire regimes can be characterized with several parameters: fire return interval (FRI), maximum fire intensity, length of the fire season, maximum fire size, and mean annual area burned [14]. Each variable is important for assessing links among climate, vegetation, and biogeochemical systems, and each can potentially be reconstructed using the three approaches listed above, *i*.*e*. sedimentary charcoal particles, dendrochronology through fire scars, and historical records, to contextualize modern fire regimes derived from remotely-sensed data [14]. FRIs indicate the average length of time for plant growth between fires, fire intensity describes the energy released by fires, the size of individual fires reflects fuel continuity and flammability, and mean burned area is an integrated metric of carbon flux from the biosphere to the atmosphere [1]. Globally, grassland biomes tend to exhibit frequent-intense-large and frequent-cool-small fire regimes, but North America does not fit this pattern, possibly due to different species composition or alternative fuel types (grasses, litter, and wood) [14]. Thus, there is a need to improve descriptions of fire regimes in grassland systems, which requires reconstructions of fires over centuries to millennia.

Long-term FRI is calculated using a statistical approach to charcoal count data over millennial time scales. A time series of charcoal pieces is separated into background charcoal and charcoal peaks [15]. Those peaks are interpreted as fire episodes, which accurately reflect the infrequent-intense-large fire regimes of some types of coniferous forests. In forested areas, extensive literature on calibration of charcoal abundance regarding the fire source [16–18], and statistical analyses of the charcoal signal (e.g. [19–21]) allow paleofire reconstruction from sedimentary charcoal to be conducted almost routinely. However, the frequency of fires in grassland systems ranges from decadal to subannual, posing a huge challenge to reconstruction of individual fire events. Indeed, the charcoal peaks, interpreted as fire events, from grassland sedimentary records are difficult to detect from the background [22]. Contrary to forested areas, only a few studies have started to calibrate charcoal count in mixed grass-tree ecosystems with modern fires [22–24]. Of those few studies, none of them reach the same conclusion about fire regime components such as fire frequency or fire intensity, as discussed in [24]. Part of the variation could be due to the broad range in size of charcoal particles considered in each study, from 3 μm to greater than 250 μm.

In non-forested environments, fuel type is an important metric to consider, and charcoal shape provides some information about fuel type. Separating woody and herbaceous charcoal was identified as a research priority in a recent global synthesis of charcoal data [25]. Categorical morphotypes have been used to characterize mixed-fuel fire regimes, but this approach has some limitations such as high variability among classification systems [26], lack of direct correspondence between fuel types and charcoal morphotypes [27], and the necessity of data reduction from multiple categories [28]. Further, Leys and collaborators [24] demonstrated that a charcoal classification scheme does not reflect the dominant vegetation cover in a mixed grass-forest environment, and may not represent these mixed fuel types accurately. An alternative metric, the averaged ratio of the width:length of charcoal pieces (W:L), provides a continuous variable that is straightforward to standardize among users, and can be directly linked to fuel type as grasses produce thinner and longer charcoal particles than woody tissue [29]. W:L ratio has been helpful in reconstructing African grassland fire regimes as the ratio reflected the surrounding landscape fuel [22]. However, there is no study in pure grassland ecosystems that has tested this ratio across a range of fire types in a sediment depositional context.

**Fig 1:**
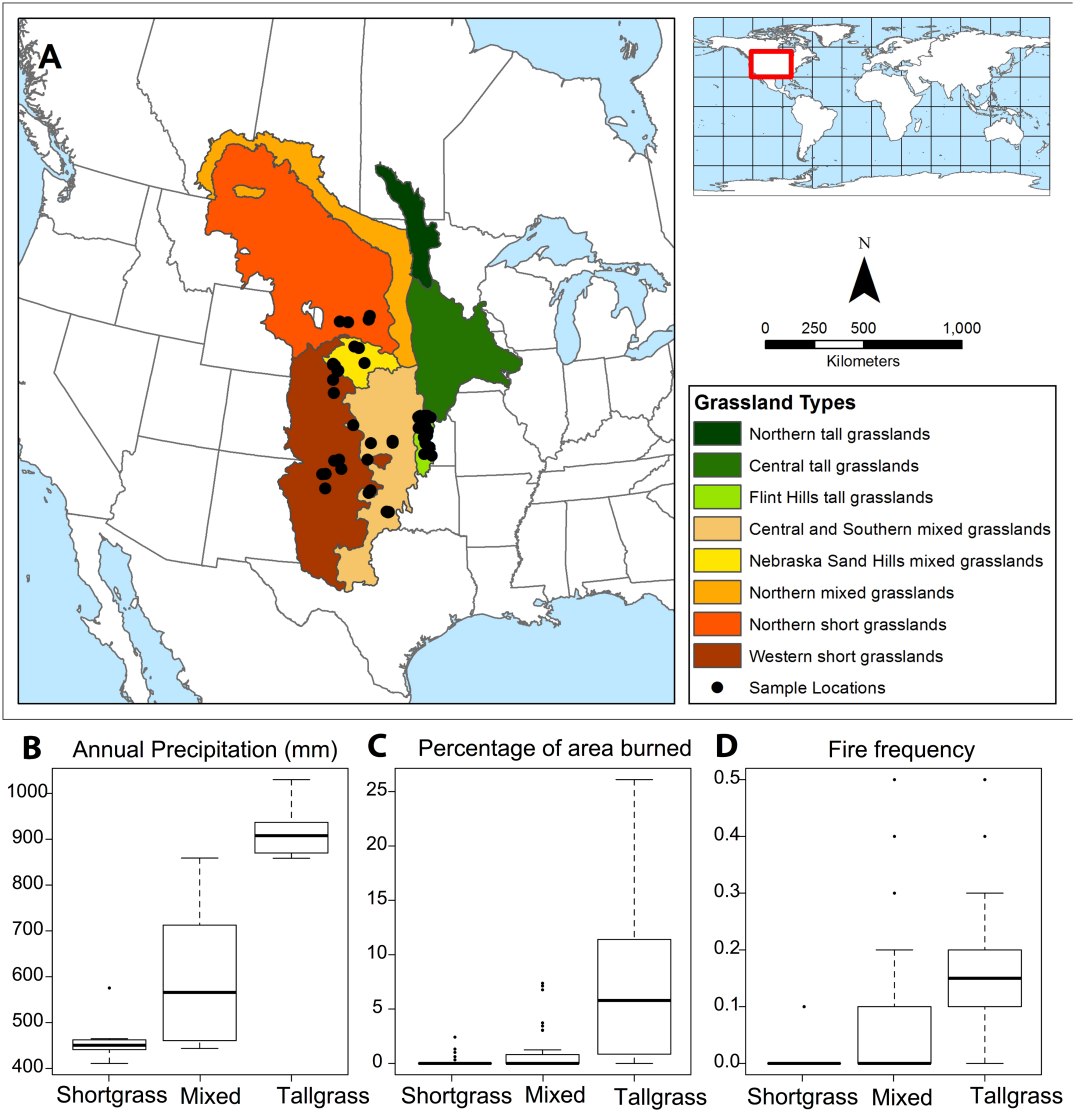
Description of the study site. **A**. Map of the 51 site locations, the grassland formations in the Great Plains. **B**, **C** and **D**. boxplots of the annual precipitation (in mm), the percentage of area burned, and the fire frequency (# of fires in ten years), respectively, of the three grassland types of the Great Plains (tallgrass, mixed grass and shortgrass prairies). Boxplots represent the mean (solid black line), first and third quartile (box limits), and 5^th^ and 95^th^ quantile of the data distribution.

**Fig 2:**
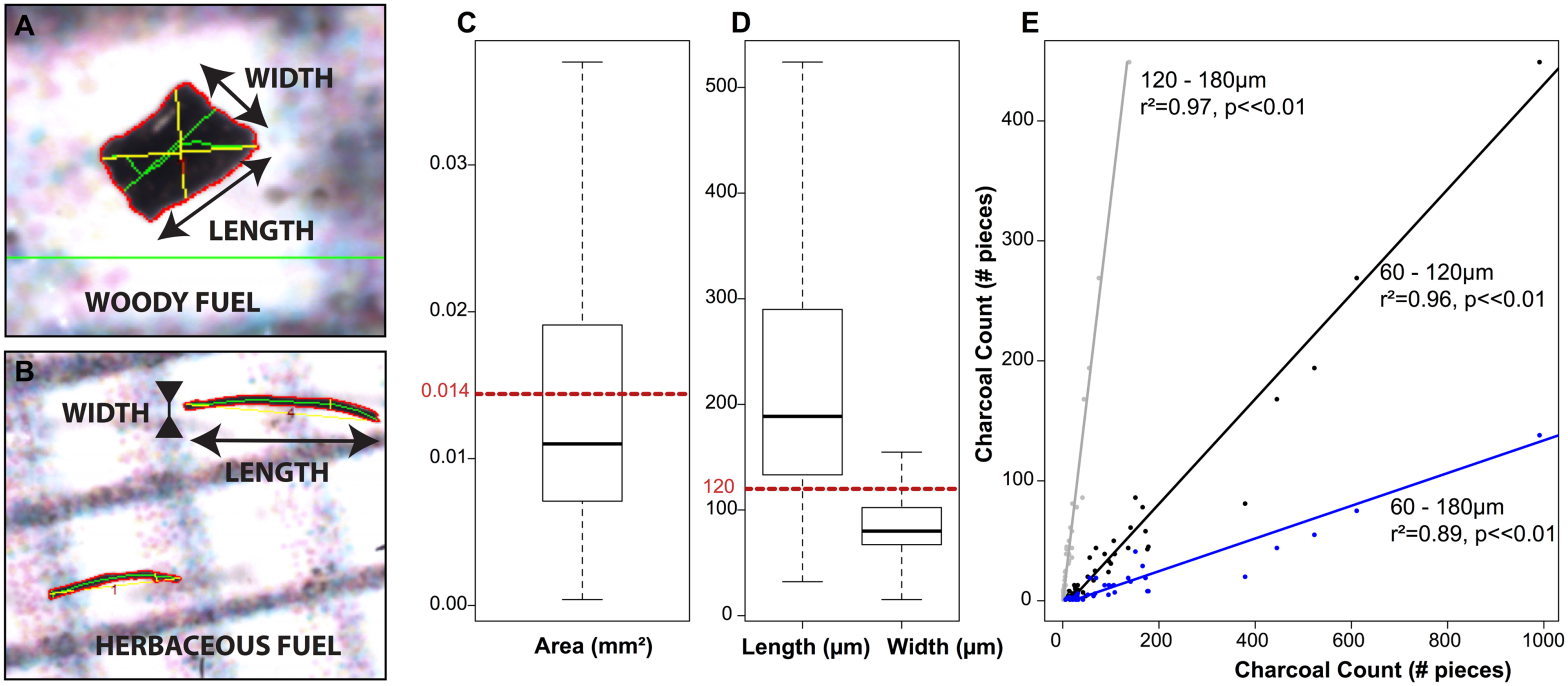
Shapes and sizes of charcoal in the Great Plains. **A** Typical shape of a woody fuel type charcoal piece; **B** Typical shape of an herbaceous fuel type charcoal piece; **C** Boxplot of the range size of area of particles from the 51 surface sediment samples. The dashed line at 0.014 mm^2^ corresponds to the area of a particle of 120x120μm. **D** Boxplots of the length and width of the charcoal particles. The dashed line is drawn at 120μm. **E** Linear regressions of charcoal count between (i) 60μm to 1 mm (x-axis) and between 120μm to 1mm (y-axis) (60–120μm); (ii) 60μm to 1mm (x-axis) and 180μm to 1mm (y-axis) (60–180μm); and (iii) 120μm to 1mm (y-axis) and 180μm to 1mm (x-axis) (120–180μm). The r-square and the p-values are indicated in the graph.

To improve reconstructions of grassland fire regimes, it is essential to assess charcoal count and shape against quantitative indices of fire regimes. Here, we present charcoal results from 51 surface sediment samples of ponds in the Great Plains of the United States. The selected sites are distributed across tallgrass, mixed grass, and short grass prairie ecosystems (Fig 1A), which are strongly linked to an east-west precipitation gradient. The shortgrass prairie is in the western, drier part of the Great Plains, the tallgrass prairie is in the eastern, wetter part of the Great Plains, with the mixed grass prairie in between (Fig 1B). The fire activity, including fire frequency and the percentage of area burned is greater in the tallgrass prairie than in the two other formations due to annual burns conducted by landholders [30] (Fig 1C–1D). The large spatial extent of the study sites is necessary to produce robust interpretations of charcoal from lacustrine and pond depositional environments, and these could eventually be used for millennial-scale fire reconstructions. This range of climate conditions and fire activity throughout the Great Plains allows us to address three research questions: 1) How can we assess the fire regime with the charcoal signal in grasslands?, 2) Do the climate or the prairie types affect the charcoal signal?, and 3) Is the fuel mainly comprised of herbaceous species? In this study, we quantified the number of charcoal pieces in sediment samples and tested its relevance as a proxy for the fire regime by examining 13 potential factors influencing charcoal count, including various fire regime components (e.g. the fire frequency, the area burned, and the fire season), vegetation cover around the sites and pollen assemblages from the same sediment samples, and climate variables. We also quantified the W:L ratio of charcoal particles (Fig 2A), to assess its utility as a proxy of fuel type in North American grassland environments by direct comparison with vegetation cover and pollen assemblages.

## Materials and methods

### Study area

The 51 surface sediment samples were collected in the Central Great Plains in 2008 for the eastern sites in the Flint Hills region, and 2011 for the other sites, from small ponds (less than 10 ha in surface area) using an Eckman dredge. Permission to sample each pond was explicitly granted by each individual landowner. No threatened or endangered species were harmed during sample collection. The Great Plains region of North America is dominated by approximately 1.3 million km^2^ of grassland vegetation. These grasslands can be further subdivided into tallgrass, mixed grass, and short grass prairie (Fig 1A), and are mainly distinguished by differences in precipitation from east to west across the region. Shortgrass prairies are found in the western (drier) portion of the Great Plains, with annual precipitation ranging from 410 to 575mm (Fig 1B) and are dominated by species such as *Buchloe dactyloides* (buffalo grass) and *Artemisia fridiga* (prairie sagewort). Tallgrass prairies are found in the eastern (wetter) portion of the Great Plains, with annual precipitation ranging from 858 to 1030mm (Fig 1B) and contain species such as *Andropogon gerardii* (big bluestem), *Sorghastrum nutans* (Indian grass), and *Ambrosia psilostachya* (western ragweed). The tallgrass prairie also contains more tree species such as *Quercus macrocarpa* (bur oak), *Juglans nigra* (black walnut), *Maclura pomifera* (Osage orange), and *Ulmus americana* (American elm). The mixed grass prairie contains a unique assortment and abundance of plant species from both neighboring tallgrass and shortgrass ecosystems, and the annual precipitation of this zone ranges from 443 to 858mm (Fig 1B).

The fire activity derived from satellite imagery data (see section “Climate and fire data” below for more details) is highest for the tallgrass prairie. Tallgrass prairie has a maximum percentage of area burned of 26% (within 5000m of the study sites) (Fig 1C) and a mean fire frequency of 0.5 fires in ten years (Fig 1D). This very high fire frequency for tallgrass prairie is partly due to private land management practices in the Flint Hills region (Fig 1A), characterized by annual, early-spring burning with heavy grazing [30]. The shortgrass prairie experiences the lowest percentage of area burned, with a maximum of 2.4% (Fig 2C), and the lowest fire frequency, with a maximum at 0.02 fires in ten years (Fig 2D).

### Charcoal data

The top 2 cm of the 51 surface sediment samples were retained from each sample. The surface sediment samples intrinsically account for taphonomic processes of transportation and deposition of charcoal into the lake [31,32]. Chronologies available from nearby sites within the Great Plains display an amount of time of approximately ten years in the top 2-cm of sediment deposition for ponds at present day (Neotoma database).

One cubic centimeter of sediment from each sample was sieved at 1mm to remove insects, and vegetation remains. No charcoal particles over 1mm were found. The samples were treated with H_2_O_2_ at 30% for 24–72 hours to remove the organic matter, and the sample was sieved with a 60μm mesh. The area of each charcoal particle, the length and width of each particle, and the total count of charcoal pieces per sample were measured with the program WinSeedle (Regent Instrument Inc., regular version 2016).

The area of charcoal has been demonstrated to limit biases on the interpretation of charcoal count, due to possible fragmentation of charcoal pieces during taphonomic processes such as transportation and deposition [32]. On the other hand, it also has been demonstrated in the Alps that big particles of charcoal can be transported long-distances [33]. We calculated the explanative power of both the count and the area of charcoal particles for reconstructions of the fire regime (*i*.*e*. the fire frequency, the area burned, and the seasonality of fire events).

W:L ratio was calculated by a simple ratio for each particle of the width to the length. We tested the relevance of this metric as a proxy of the fuel types with the vegetation cover and the pollen assemblages.

Experimentation suggests that large charcoal pieces (i.e. >100 μm, [34]) allow a local reconstruction of fire history due to limited charcoal transportation from the source area [17,35]. On the other hand, regional fire histories are revealed by smaller particles (*e*.*g*. <180 μm in [36] or <50 μm in [37]). We sorted the count of charcoal pieces into three size categories: (i) 60μm to 1 mm, (ii) 120μm to 1mm, and (iii) 180μm to 1mm to test the source of charcoal particles.

### Vegetation data: Pollen and satellite imagery

To measure the landcover surrounding each pond, a pre-classified vegetation dataset was acquired from the National Gap Analysis Program (United States Geological Survey, 2011). This dataset has a 30-m spatial resolution and was classified using multi-season satellite imagery (Landsat ETM+) The percent cover of each landcover type was extracted within 1060-m and 5000-m buffers of each pond in GIS. A buffer distance of 5000-m was used because this distance has been found to be the relevant source area of charcoal in the tallgrass prairie [24], although the authors did not test the source area of charcoal between 500 m and 5000 m due to the overlapping buffer distances of their depositional environments. A buffer distance of 1060-m was used because this distance has previously been found to be the relevant source area for pollen samples from ponds of similar size in this region [38]. We used both buffer distances to be as comprehensive as possible, since relevant source area for charcoal from lacustrine samples has not been as well-studied as pollen. Five natural landcover types (Forest & Woodland, Nonvascular & Sparse Vascular Rock Vegetation, Open Water, Semi-Desert, and Shrubland & Grassland) and four cultural (human-induced) landcover types (Agricultural, Developed & Other Human Use, Introduced & Semi Natural Vegetation, and Recently Disturbed or Modified), were found within these buffers. Shrubland & Grassland was the most abundant type, comprising 75% of the landcover surrounding the ponds (on average). Each 30-m pixel classified as Shrubland & Grassland contains less than 10% tree cover and the remaining 90% consists of native shrubs, herbs, and grasses. The second most abundant type, Agricultural, consists of row crops or other planted crops. Forest & Woodland (less than 5% of the cover within the buffers, on average) is dominated by temperate broadleaf or needleleaf trees.

Pollen data from the same sediment samples at each site were used to calculate two additional vegetation metrics: the ratio of arboreal to non-arboreal pollen (AP/NAP), and the ratio of *Ambrosia* to *Artemisia* pollen. The AP/NAP pollen ratio is not linearly transferrable to vegetation cover because plant species vary in pollen productivity [38]. However, the ratio can be used as a relative metric of woody versus herbaceous vegetation across the sites. The *Ambrosia*:*Artemisia* ratio can be used to distinguish between tallgrass and shortgrass prairie vegetation. A high value of *Ambrosia*:*Artemisia* indicates a vegetation assemblage dominated by *Ambrosia* and higher precipitation in comparison with a low value of this ratio, which indicates a dominance of *Artemisia* in the vegetation assemblage and lower precipitation [39]. *Ambrosia* species are more abundant in the eastern tallgrass prairies, where the precipitation is higher than in the western shortgrass prairies, which support more *Artemisia* species.

### Climate and fire data

Gridded 30-year normal temperature data (average mean, maximum, and minimum for each month and annually) and precipitation data (total monthly and annual) were acquired from the PRISM Climate Group (PRISM 2015, http://prism.oregonstate.edu). These data cover the period from 1981–2010 and have a spatial resolution of 4 km. The temperature values (average mean, maximum, and minimum for each month and annually) and precipitation values (total monthly and annual) at each sample location were extracted in ArcGIS using standard “extract by location” tools.

Fire data were acquired as a shapefile from the Monitoring Trends in Burn Severity project (www.mtbs.gov), which has recently mapped fires in the United States that occurred between 1984–2014, based on Landsat satellite imagery with a 30-m spatial resolution. This dataset includes the spatial boundaries, date, and type (wildfire, prescribed) of each fire. We extracted the area burned within the 1060-m and 5000-m buffers around each site between the years 2000–2009 for the Flint Hills samples, and the years 2003–2012 for the remaining samples, which correspond with the ten years prior to the collection year of the sediment (2009 for the Flint Hills samples, and 2012 for the remaining samples). In this study, fire activity surrounding each sample site was calculated as total area cumulatively burned during the 10 years prior to sampling. There is high variability in fire activity among sites, ranging from never burned during that time to 172 km^2^ cumulatively burned, equal to 26% of area burned within 5000m radius of the site.

### Statistical analyses

Linear regressions were calculated from the count of charcoal pieces between (i) 60μm to 1 mm and between 120μm to 1mm; (ii) 60μm to 1mm and 180μm to 1mm; and (iii) 120μm to 1mm and 180μm to 1mm for different source areas, *i*.*e*. a regional fire history for the smallest ones or a more local fire history for the biggest ones, as explained in the literature [24]. R-square and p-values were calculated for each regression with R software [40].

A principal component analysis (PCA) was conducted to decipher the relationship among the 15 most abundant pollen taxa, the ratio of *Ambrosia* to *Artemisia*, the AP/NAP, the W:L ratio, and the area of charcoal particles. In order to have one value of charcoal metrics per sample, we calculated the mean of W:L ratio values for each sample and the sum of charcoal particles for each sample.

Random forest analyses were used to assess the explanatory power of the 13 environmental parameters on the charcoal count, the area of charcoal, and the W:L ratio of charcoal pieces. Random forest is a based on a decision-tree method that allows for both quantitative and categorical variables [41], and it has been applied to analysis of charcoal data in previous studies [24]. The importance of each parameter is estimated by the reduction of the predictive skill on the out-of-bag samples, and expressed as mean standard error (MSE). A large positive value of MSE indicates that the parameter tested is highly predictable, a null value indicates that the parameter is not predictable, and a negative value indicates that adding the parameter decreases the prediction ability.

## Results and discussion

### Practical recommendations for charcoal analysis in grassland systems

#### Charcoal size categories

Overall, sizes of charcoal particles across the 51 study sites are small, with 75% of the charcoal particles less than 120μm, and half of the particles less than 93μm (Fig 2C, S1 Fig). Large charcoal particles (over 120μm in size) represent only ~25% of the total charcoal particles, averaging 29 particles per sample (Fig 2C). Comparing the distribution of charcoal among sites, there is an important difference in the number of particles for each size: 4% of the samples have no charcoal particles over 120μm (20% for charcoal >180μm), and 25% of the samples have lower than 6 particles (50% for charcoal >180μm) (S2 Fig).

However, the results show a strong correlation between particles from 60μm to 1mm and from 120μm to 1mm (r^2^ = 0.96), between particles from 60μm to 1mm and from 180μm to 1mm (r-square = 0.89), and between particles from 180μm to 1mm and from 120μm to 1mm (r-square = 0.97) (Fig 2E), indicating that there is no change in transport, deposition, or preservation among these three sizes.

Samples sieved with a mesh size of 60μm have higher charcoal counts than those sieved with a 120μm mesh as used in forested environments [42], or 180μm mesh as used in the grasslands of Mongolia [43]. It is likely that a mesh size under 60μm could produce an even higher charcoal particle count, but quantification and image acquisition would become bigger issues if the lithology includes dark minerals, or the pixel size resolution of the camera or the image analyzing software is too large. When analyzing a long-term charcoal record, samples with values of 0 can limit statistical power. Therefore, we recommend counting all charcoal particles over 60μm in grasslands and mixed grass-forest environments to increase the number of samples with useful data.

#### Charcoal count and area of charcoal particles reflect the local area burned

Most paleofire studies use the number of charcoal particles to reconstruct fire regime, *i*.*e*. the fire frequency or the biomass burned [16,44], but a few studies have shown that the area of charcoal pieces is a better proxy of fire regime because it accounts for fragmentation processes of thin and fragile charcoal particles, which could happen during the transport or deposition of charcoal [32,45,46]. Our results show a strong correlation between the count and the area of the charcoal particles (r-square = 0.94, figure not shown), which indicates that the count and the area of charcoal pieces are providing the same information and that there are few fragmentation processes in these grassland environments. However, this result needs to be tested on a longer time scale, including during past climate events that could have changed the fragmentation processes.

**Fig 3:**
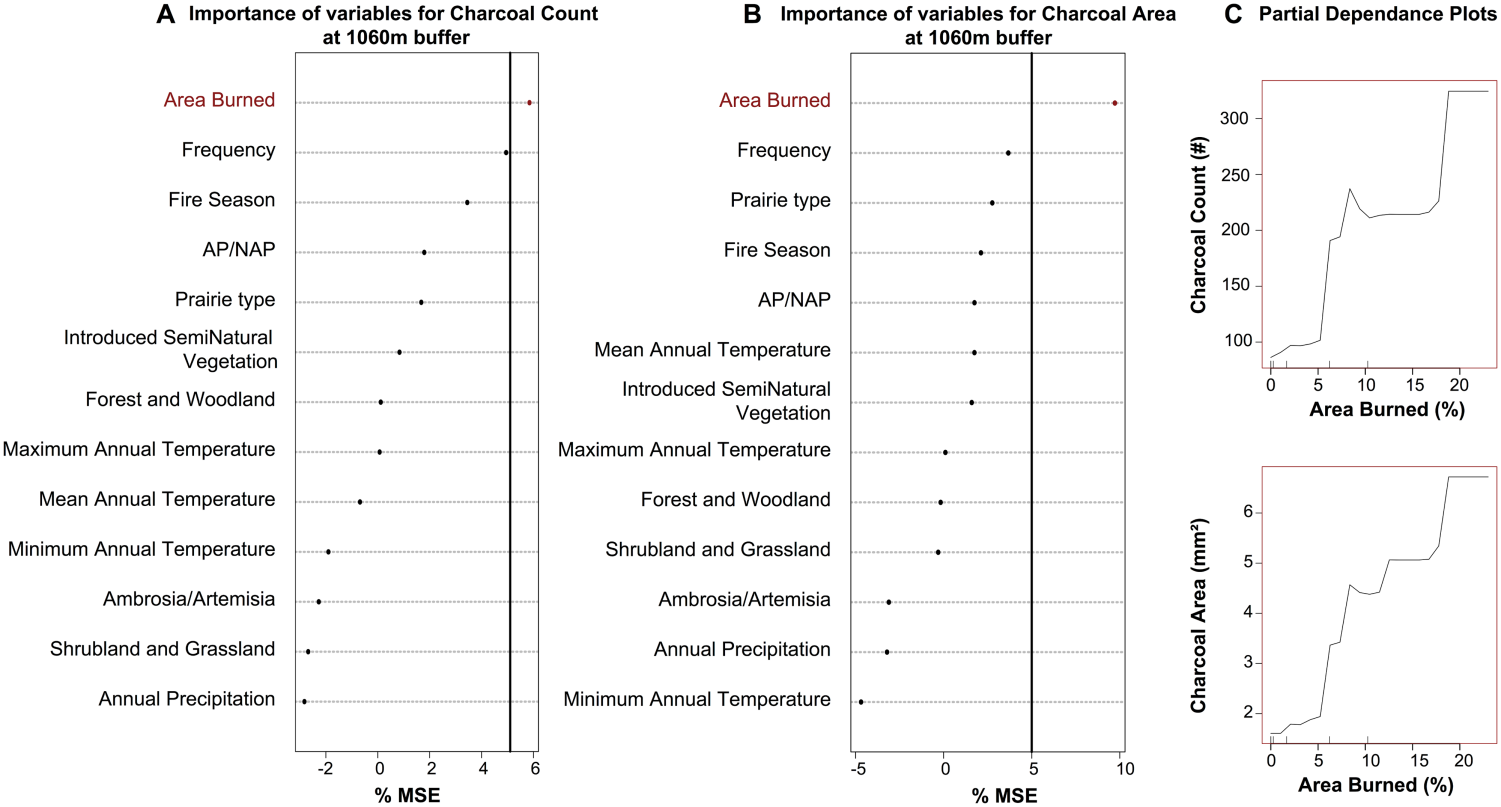
Random forest analyses of 13 explanatory factors at a 1060m buffer. Random forest results expressed in percentage of mean standard error (%MSE) of **A** the charcoal count (Char Count), and **B** the sum of the particles’ area per sample (Char Area). **C** For each random forest analysis, the partial plot of the factor explaining more than 5% of the variance.

Random forest analysis indicates that the area burned within 1060m of the depositional environment explains charcoal area and charcoal count, at 9.7% and 5.8% respectively (Fig 3). The partial plots indicate that the area burned is positively correlated with both the area and the count of charcoal pieces, which indicate that large burnt areas produce more charcoal than small burnt areas. Area burned was also correlated with charcoal count in a North American tallgrass prairie, but at larger spatial extents than 1000m, which was not tested in [24], due to the a significantly smaller spatial extent than the current study.

The other fire variables we analyzed in this study—fire frequency and fire season—do not explain either of the two charcoal variables—charcoal count, and the area of charcoal pieces. In addition, neither climate variables nor land cover variables significantly correlate with charcoal area or charcoal count. We thus encourage the interpretation of charcoal count or charcoal area variations through time as a change of area burned. However, it is important to note that regional- to continental-scale spatial datasets delineating burned areas (such as the dataset used in this paper) often omit many grassland burns because the temporary nature of their scars precludes detection in satellite imagery and image classification [47]. It is possible that stronger connections could be made between charcoal variables and fire variables through the use of a higher-resolution burn dataset. Currently, there is no dataset to our knowledge that effectively captures both the temporal and spatial resolution of North American grassland fires. As we further study the fire regimes of grassland systems [14] to understand human and climatic influences on fire behavior, long-term records can be of great value.

#### Charcoal shape (W:L ratio as a proxy of fuel types)

In our study, there is a clear difference between the width and the length of charcoal particles, with more than 75% of the particles displaying a length over 120μm, or a width under 100μm (Fig 2D), which indicates overall low values of W:L ratio. The W:L ratio is useful for assessment of fuel types, either herbaceous or woody, with higher values indicating a fuel type dominated by woody species, and lower values indicating a fuel type dominated by grass or other herbaceous species [29].

**Fig 4:**
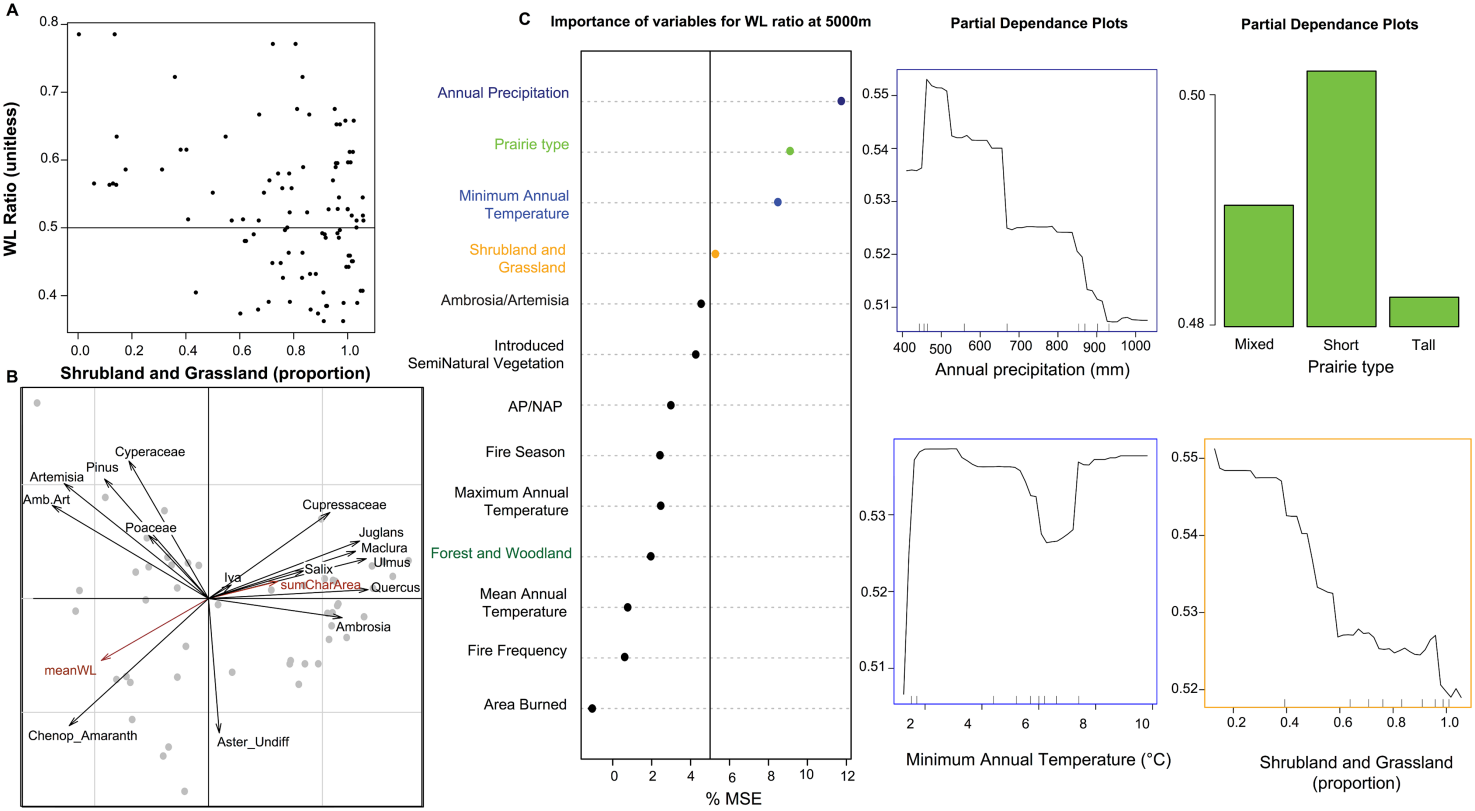
Relationships among the width to length ratio (W:L ratio) of charcoal particles and the environmental parameters. **A** Scatter plot between W:L ratio averaged by site and the proportion of Grassland and Shrubland on the landscape, within a 5000m buffer from the depositional environment. The line corresponds to a W:L ratio of 0.5. **B** Principal component analysis of the 15 most abundant pollen taxa present in the 51 surface sediment samples, the mean value of W:L ratio for each site (meanW:L), and the sum of area of particles for each site (sumCharArea). Amb.Art is the ratio of *Ambrosia* to *Artemisia* pollen, Chenop_Amaranth for Chenopodiaceae/Amaranthaceae pollen, and Aster_Undiff for undifferentiated species of Asteraceae pollen. Axis 1 explains 27% and axis 2 explains 13%. **C** the random forest analysis of the 13 explanatory factors of the W:L ratio within a 5000m buffer from the depositional environment, and the partial dependence plots for the factors explaining more than 5% of the variance. The y axes of the partial dependence plots correspond to the W:L ratio (unitless). AP/NAP for the ratio of arboreal pollen to non-arboreal pollen.

However, vegetation cover does not directly reflect fuel types, so the relationship between W:L ratio and woody cover is not straightforward. For example, non-forested landscapes were associated with a width/length ratio <0.5 in a study in the Afrotropics [22]. In our study, the mean W:L ratio varies among sites from 0.3 to 0.8 for a charcoal size of 60μm (from 0.05 to 0.74 for 120μm and from 0.05 to 0.7 for 180μm), and has lower values with a larger proportion of shrubland and grassland around the depositional environment (Fig 4A). While not statistically significant, no sites surrounded by less than 40% grassland and shrubland have a W:L ratio lower than 0.5 (Fig 4A). For sites surrounded by more than 40% grassland and shrubland, 50% of the W:L ratio values are below 0.5. Thus, the W:L ratio of 0.5 found in the tropics also appears, in our study, to be a robust threshold for interpretation of the fuel type as composed at least 40% by grassland cover.

The W:L ratio values should be examined in other non-forested environments, mixed fuel environments, and across temporal transitions in vegetation. For example, the branched morphotypes described by Jensen et al. [27] include thin and long charcoal morphotypes, but are derived from woody fuel types.

### Climate influence on grassland fires

The relationships among charcoal metrics and vegetation composition were analyzed in more detail with a principal component analysis of charcoal and pollen in the sediment at each site. Principal component axis 1 explains 26%, and axis 2 explains 12% of the variance in all variables (Fig 4B). PC 1 is significantly influenced by the area of charcoal particles and by the pollen percentages of several woody taxa and *Ambrosia*. PC 2 is significantly influenced by pollen from Chenopodiaceae and Amaranthaceae, Poaceae, and *Pinus*, and the W:L values. Sites from the shortgrass prairies tend to cluster in the top left quadrat (with Poaceae, *Artemisia*, *Pinus*, and Cyperaceae) while tallgrass prairie sites are found on the right side of the figure, with positive PC1 scores and high amounts of *Ambrosia*, *Quercus*, *Juglans*, *Maclura*, *Ulmus*, and Cupressaceae pollen. Chenopodiaceae and Amaranthaceae are separate from the other pollen taxa along PC2. The PCA results demonstrate: 1) tallgrass and shortgrass prairies have distinctly different pollen signatures, and 2) higher charcoal area and lower W:L ratios are associated with sites from tallgrass vegetation.

Climate variables at each site are also linked with the W:L ratio in addition to pollen composition, as revealed by a second multivariate analysis using a random forest approach (Fig 4C). Four factors significantly influenced the W:L ratio among sites: mean annual precipitation (10.1%), prairie type (8.9%), annual minimum temperature (7.7%), and the proportion of shrubland and grassland within 5000 m of the depositional environment (6.5%). Annual precipitation and the proportion of shrubland and grassland are both negatively correlated with the W:L ratio, indicating an increase of herbaceous or grass fuel types with increasing precipitation and increasing grassland and shrubland cover. Minimum annual temperature is positively correlated with the W:L ratio, which indicates that sites with cool minimum annual temperatures are recording high amounts of herbaceous or grass fuel types. Finally, sites from the tallgrass prairie type are associated with low values of the W:L ratio, indicating that tallgrass prairie produces more elongated charcoal particles than shortgrass or mixed grass prairies. The three most explanatory factors are the same in the random forest analysis for variables calculated with distances of 1060m around each site (S3 Fig). None of the included fire variables significantly influenced the W:L ratio.

Grassland fires in the Great Plains region vary in their timing, frequency, severity, and area burned. Fire management approaches need to account for this spatial variability both for prescribed fires and for fire suppression efforts. We identified regional differences in how fires currently burn, and how those are represented in charcoal deposited in sedimentary basins. Climate variables can potentially influence grassland fires through fuel limitation [48] and fuel condition [49]. In our study, annual precipitation (ranging from 410 to 1030 mm, Fig 1B) and temperature (ranging from 8.39 to 16.23°C) are the two most predictive climate variables of the charcoal W:L ratio, which indicates fuel type. In the Great Plains, Morris [39] and Commerford et al. [11] have demonstrated that mean annual precipitation is strongly linked to the differences in the dominant grassland plant taxa, with *Artemisia* species more common in the drier shortgrass prairies, and *Ambrosia* species more common in the wetter tallgrass prairies. The precipitation gradient is also strongly correlated with the biomass productivity of grass species [9], with wetter conditions providing more fuel for fires. Taken together, the increase of precipitation and the range of minimum temperature, both linked to an increase in biomass and associated with tallgrass prairie assemblages, explained higher values of the W:L ratio.

There are mixed results in this study about the relationship between charcoal area and vegetation type. Higher charcoal area is related to woody plant pollen like *Quercus*, Cupressaceae, *Ulmus*, or *Juglans*—dominant plants in the tallgrass prairie but not in the shortgrass or mixed-grass prairie—which seems to indicate that higher charcoal area is related to higher amounts of biomass. However, the random forest analysis did not show that the prairie types are a strong explanative parameter of the charcoal area (Fig 3B). Even if the area of charcoal seems to be indirectly correlated with more biomass, our results did not support any significant direct relationship between the area of charcoal and the prairie types, or the biomass burned.

### Vegetation influence on grassland fires

Although vegetation composition is correlated with climate at the regional scale, there is an independent influence of vegetation composition on charcoal metrics, perhaps operating at local scales. In our analyses, the W:L ratio is negatively correlated with metrics of woody vegetation, which is initially counterintuitive since lower values of W:L ratio have been interpreted as indicating a higher proportion of herbaceous and grass fuel types. However, tallgrass prairies have a relatively high proportion of woody vegetation cover as indicated by pollen from *Quercus*, Cupressaceae, *Ulmus*, *Juglans*, and other woody taxa [50]. The climate signal described above, with wetter tallgrass prairies exhibiting less fuel limitation, may be embedded in the vegetation composition variables. This conclusion is supported by the random forest analysis, where the annual precipitation and the prairie types are the most important variables explaining the W:L ratio.

Alternatively, interpretations of the W:L ratio could be simply a matter of spatial heterogeneity in fuel flammability, specifically differences between the actual fuel burned during a fire and the dominant vegetation types around the site. A semi-forested landscape can experience low severity fires burning essentially the herbaceous and grass species, as has already been demonstrated in a tropical environment [22] and a temperate savanna environment [28]. In this situation, the W:L ratio is providing essential information about the type of vegetation that burns in the region. Here, fires consume the herbaceous layer, thereby reducing competition for the unburned tree layer and allowing it to expand despite high fire frequency [51].

## Supporting information

S1 Fig Charcoal area and width to length ratio per sites. The charcoal area is expressed in mm^2^, and the width to length ratio (W:L ratio) is unitless. GP = sites in the short and mixed grass prairies of the Great Plains. FH = sites in the Flint Hills, tallgrass prairie of the Great Plains.
(TIFF)

S2 Fig Charcoal count by samples for the three different size ranges. From top to bottom: from 60μm to 1mm, from 120μm to 1mm, and from 180μm to 1mm.
(TIFF)

S3 Fig Random forest analyses of 13 explanatory factors at a 1060m buffer of the width to length ratio of charcoal particles. Random forest results in percentage of mean standard error (%MSE), and the partial plots of the factor explaining more than 5% of the variance.
(TIFF)
