# Supplementary figures and images for: Reconstructing grassland fire history using sedimentary charcoal: Considering count, size and shape
Source: PLoS One. 2017 Apr 27;12(4):e0176445. doi: 10.1371/journal.pone.0176445 (PMC5407794; doi:10.1371/journal.pone.0176445)

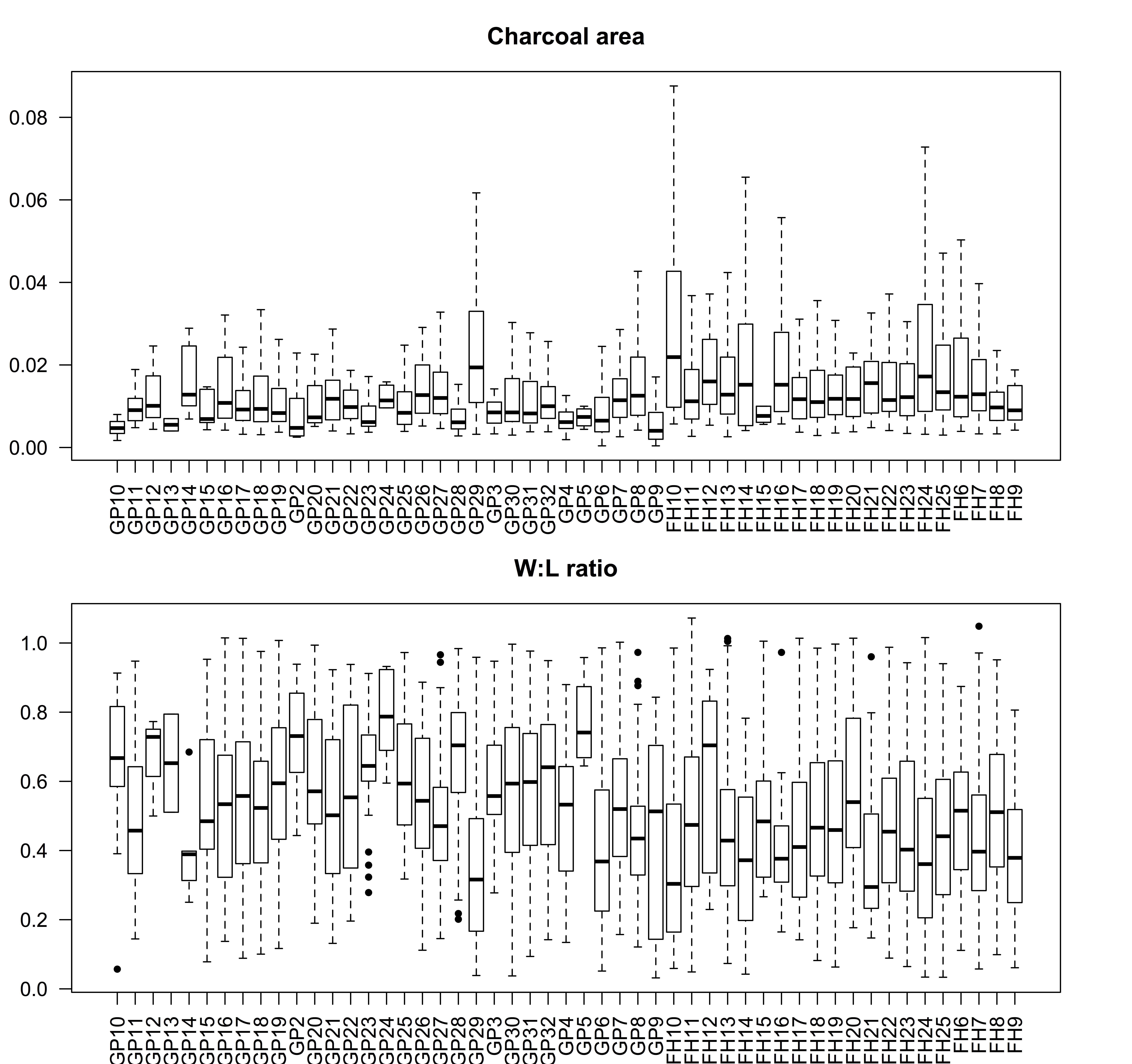

Supplement: S1 Fig — The charcoal area is expressed in mm2, and the width to length ratio (W:L ratio) is unitless. GP = sites in the short and mixed grass prairies of the Great Plains. FH = sites in the Flint Hills, tallgrass prairie of the Great Plains. (TIFF) [file pone.0176445.s001.tiff]

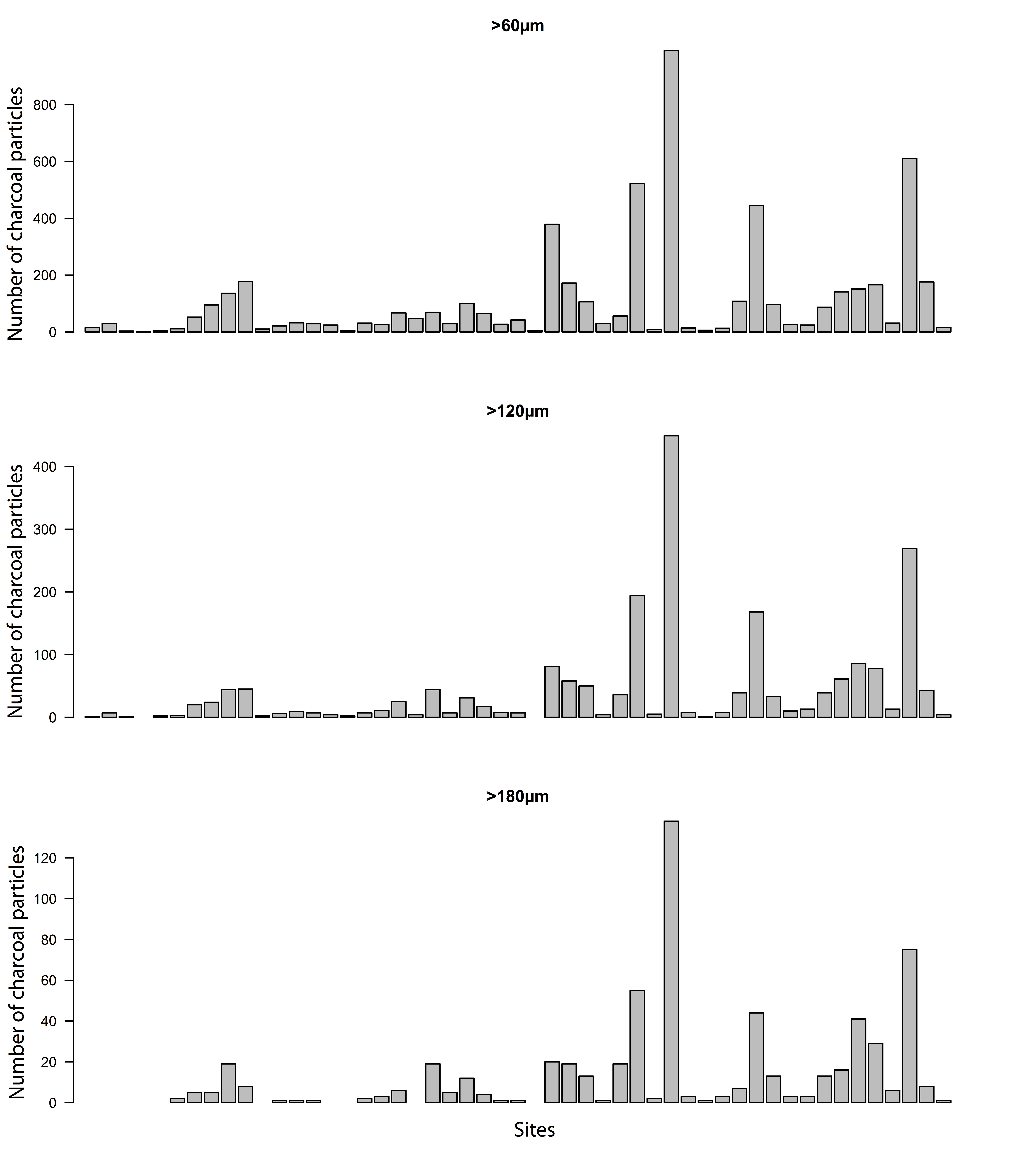

Supplement: S2 Fig — From top to bottom: from 60μm to 1mm, from 120μm to 1mm, and from 180μm to 1mm. (TIFF) [file pone.0176445.s002.tiff]
